# A Pentapeptide with Tyrosine Moiety as Fluorescent Chemosensor for Selective Nanomolar-Level Detection of Copper(II) Ions

**DOI:** 10.3390/ijms21030743

**Published:** 2020-01-23

**Authors:** Krzysztof Żamojć, Dominik Kamrowski, Magdalena Zdrowowicz, Dariusz Wyrzykowski, Wiesław Wiczk, Lech Chmurzyński, Joanna Makowska

**Affiliations:** Faculty of Chemistry, University of Gdańsk, Wita Stwosza 63, 80-308 Gdańsk, Poland; dominik.kamrowski@gmail.com (D.K.); magdalena.zdrowowicz@ug.edu.pl (M.Z.); dariusz.wyrzykowski@ug.edu.pl (D.W.); wieslaw.wiczk@ug.edu.pl (W.W.); lech.chmurzynski@ug.edu.pl (L.C.); joanna.makowska@ug.edu.pl (J.M.)

**Keywords:** Cu^2+^ ions, fluorescent chemosensor, pentapeptides, fluorescence quenching

## Abstract

Herein, we have investigated principally with the use of UV and fluorescence (steady-state and time-resolved) spectroscopy the interactions between selected pentapeptides with tyrosine residue (EYHHQ, EHYHQ, EHHQY, and KYHHE) and various metal ions (Cu^2+^, Mn^2+^, Co^2+^, Ni^2+^, Zn^2+^, Cr^3+^, Cd^2+^, Ag^+^, Pb^2+^, Sr^2+^, Ba^2+^, Ca^2+^, Mg^2+^, Al^3+^, Fe^2+^, and Ga^3+^) in order to establish the relationship between the position of a tyrosine residue in the peptide sequence and the metal ion-binding properties. Among the peptides studied, EHYHQ was evaluated as an efficient and selective ligand for developing a chemosensor for the detection of copper(II) ions. While significant fluorescence emission quenching was observed for that peptide in the presence of Cu^2+^ cations, other metal cations used at the same and at considerably higher concentrations caused a negligible change of the fluorescence emission spectrum, indicating a high selectivity of EHYHQ for Cu^2+^ ions. Under optimum conditions, fluorescence intensity was inversely proportional to the concentration of Cu^2+^ ions. The limit of detection of Cu^2+^ ions with the use of EHYHQ was determined at the level of 26.6 nM. The binding stoichiometry of the complexes of the studied peptides with Cu^2+^ ions was evaluated spectrophotometrically and fluorimetrically (as in the case of EHYHQ confirmed by mass spectrometry) and found to be 1:2 (Cu^2+^-peptide) for all the investigated systems. Furthermore, the stability constant (*K*) values of these complexes were determined. The reversibility of the proposed Cu^2+^ ions sensor was confirmed, the pH range where the sensor acts was determined, while its analytical performance was compared with some other reported recently fluorescent sensors. The mechanism of the interactions between EHYHQ and Cu^2+^ was proposed on the basis of NMR spectroscopy investigations.

## 1. Introduction

In recent years, there has been a particular emphasis on the development of new highly selective molecular sensors for cations with biological interest (such as Na^+^, Ca^2+^, Cu^2+^, and Zn^2+^) because of their potential applications to clinical biochemistry and environmental research [1]. Among these ions, the Cu^2+^, ion as the third most abundant essential transition metal in a human organism [2], plays a significant role in a variety of fundamental physiological processes (many proteins use Cu^2+^ ion as a cofactor for electron or oxygen transport as well as a catalyst for oxidation–reduction reactions [3]). The imbalance in the level of free copper ions can have a pernicious influence on biological systems—their deficiency is the symptom of anemia [4]. On the other hand, when the levels of Cu^2+^ ions exceed cellular needs (the concentration of copper in blood is expected to lie between 11.8 and 23.6 μM [5]), they catalyze the generation of reactive oxygen and nitrogen species that can damage lipids, nucleic acids, and proteins [6]. Furthermore, disorders in cellular homeostasis of Cu^2+^ ions have been proven to result in various diseases, such as Alzheimer’s disease, Parkinson’s disease, prion diseases, or amyotrophic lateral sclerosis [7]. Because of its wide applications, Cu^2+^ ion is also attributed as a significant metal pollutant. The US Environmental Protection Agency set the limit of copper in drinking water at 1.3 ppm (∼20 μM) [6].

Considering all the above reasons, there has been a growing interest in the analytical detection of Cu^2+^ ions using, for example, colorimetry [8,9], spectrophotometry [10], laser ablation inductively coupled plasma mass spectrometry [11], flame atomic absorption spectrometry [12], and voltammetry [13]. Although all these methods provide relatively low limits of detection and wide concentration ranges, many of them are destructive techniques that require the use of expensive equipment, making them not adaptable for online monitoring. Therefore, the development of fluorescent chemosensors (due to high sensitivity of fluorescence spectroscopy, easy operation, visual simplicity [14], instantaneous response, real-time detection [15], and a variety of fluorophores [16]) for the detection of Cu^2+^ ions is of great importance [17]. On the other hand, the availability of a variety of fluorophores and the selectivity of such fluorescent probes still remain a great challenge, as many recently reported chemosensors for cations demonstrated a change in their fluorescent features after binding with more than one ion [18,19,20].

Studies of the complexes of amino acids and peptides with metal ions have been performed with a main goal to better understand transition metal complexes in proteins [21,22]. For instance, it has been shown that several ions, e.g., Cu^2+^ and Zn^2+^, are involved in the amyloid-β peptide aggregation process, which presumably plays a main role in Alzheimer’s disease [23]. From among all amino acids present in the amyloid-β (1–42) peptide sequence, histidine and tyrosine side chains are proven to have a distinct affinity for Cu^2+^ and other metal cations [24]. The main goal of our work was to investigate the interactions between selected pentapeptides with a tyrosine moiety including EYHHQ, EHYHQ, EHHQY, and KYHHE, where E denotes glutamate, Y tyrosine, H histidine, Q glutamine, K lysine (structures of these pentapeptides are shown in Figure S1 in the Supplementary Materials), and a list of chosen metal ions (Cu^2+^, Mn^2+^, Co^2+^, Ni^2+^, Zn^2+^, Cr^3+^, Cd^2+^, Ag^+^, Pb^2+^, Sr^2+^, Ba^2+^, Ca^2+^, Mg^2+^, Al^3+^, Fe^2+^, and Ga^3+^) to evaluate the relationship between the position of a tyrosine residue in the pentapeptide sequence and the metal ion-binding properties.

## 2. Results and Discussion

**Figure 1:**
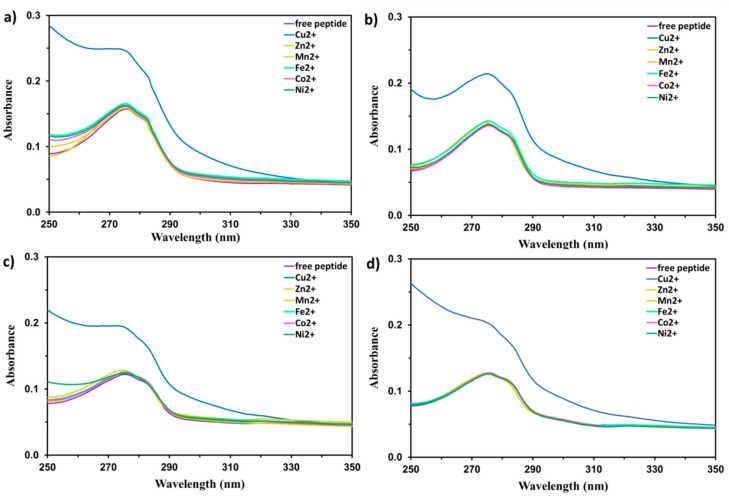
The UV absorption spectra of EYHHQ (**a**), EHYHQ (**b**), EHHQY (**c**), and KYHHE (**d**) (0.1 mM in 5 mM MES buffer, pH 6.0) in the absence of any ions (free peptide) as well as in the presence of Cu^2+^ and other cations (80 µM, molar ratio 1:0.8) including Mn^2+^, Fe^2+^, Co^2+^, Ni^2+^, and Zn^2+^.

In order to establish the metal ion-binding properties of the studied peptides, UV absorption spectra of 0.1 mM EYHHQ, EHYHQ, EHHQY, and KYHHE in 5 mM MES buffer (pH 6.0) were recorded in the absence and in the presence of 80 μM of various metal ions (molar ratio 1:0.8), including Cu^2+^, Mn^2+^, Co^2+^, Ni^2+^, Zn^2+^, Cr^3+^, Cd^2+^, Ag^+^, Pb^2+^, Sr^2+^, Ba^2+^, Ca^2+^, Mg^2+^, Al^3+^, Fe^2+^, and Ga^3+^, and are shown in Figure 1. It can be observed that the addition of only Cu^2+^ ions (80 μM) promoted a 1.5× enhanced response at 275 nm and even higher at <275 nm compared to other metal ions. This can also be observed as a rapid (a few seconds) response. A noticeable change in the shape of the spectra and intensity of the absorbance are arguments supporting the formation of a new chemical species. As no other metal ion/peptide systems showed any significant changes in their absorption features compared to that of Cu^2+^ as well as taking into consideration an evidenced stronger affinity of various peptides for Cu^2+^ ions (when compared to other metal ions) [25,26,27], we have assumed the observed result for Cu^2+^ to have arisen from the chelation of Cu^2+^ ions with the peptides under study [28,29,30,31]. Furthermore, no shift of the band with a maximum at 275 nm (as can be observed, for example, in Figure 1b) indicates that a reformed new excited electronic state of tyrosine in a complex is similar to the one in a ligand alone [32]. It is noteworthy to mention that an increase in the absorbance was observed until the peptides were saturated at approximately 0.5 molar equivalent of added Cu^2+^ and that subsequent addition of excess Cu^2+^ ions produced no significant changes in UV spectra (details will be described further and shown in Figure 3).

**Figure 2:**
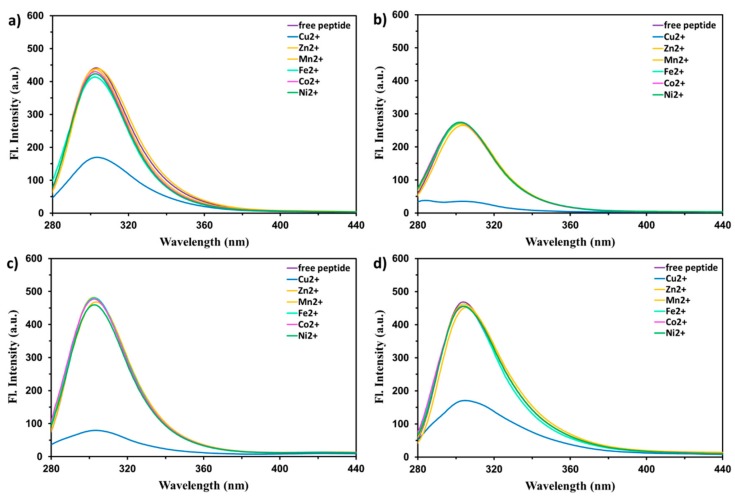
The fluorescence emission spectra of EYHHQ (**a**), EHYHQ (**b**), EHHQY (**c**), and KYHHE (**d**) (0.1 mM in 5 mM MES buffer, pH 6.0) in the absence of any ions (free peptide) as well as in the presence of Cu^2+^ and other cations (80 µM, molar ratio 1:0.8) including Mn^2+^, Fe^2+^, Co^2+^, Ni^2+^, and Zn^2+^.

**Figure 3:**
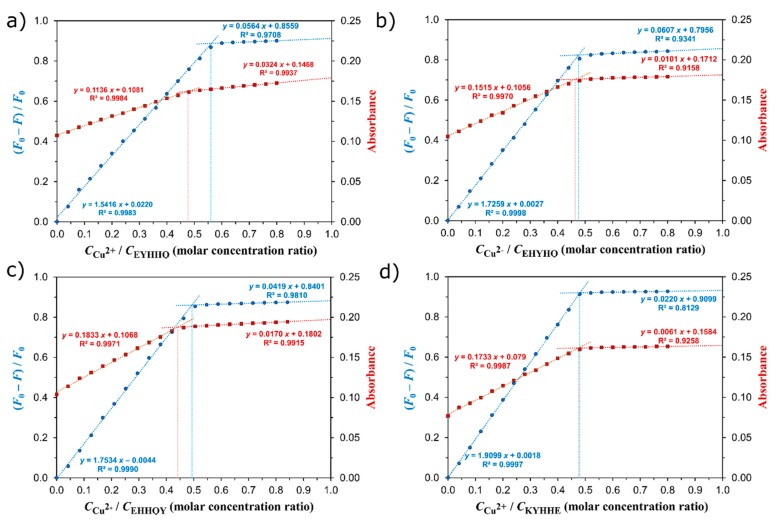
The plots of $\frac{F_{0}\;-\;F}{F_{0}}\;$(blue) and absorbance measured at 275 nm (red) against the metal ion to peptide molar concentration ratio$\;\left(\frac{c_{{\mathrm{Cu}}^{2+}}}{c_{\mathrm{peptide}}}\right)$ for EYHHQ (**a**), EHYHQ (**b**), EHHQY (**c**), and KYHHE (**d**); c_peptide_ = 0.1 mM: Line–line intersections represent the stoichiometry of the formed Cu^2+^-peptide complexes. F_0_ is fluorescence intensity (measured at 305 nm, λ_ex_ = 275 nm) of the peptide in the absence of Cu^2+^ ions; F is fluorescence intensity (measured at 305 nm, λ_ex_ = 275 nm) of the peptide at any given concentration of Cu^2+^ ions.

To further explore the selectivity of the studied peptides toward metal ions, their fluorescence emission spectra were also recorded upon excitation at 275 nm in the presence of 80 μM of various metal ions (molar ratio 1:0.8), including Cu^2+^, Mn^2+^, Co^2+^, Ni^2+^, Zn^2+^, Cr^3+^, Cd^2+^, Ag^+^, Pb^2+^, Sr^2+^, Ba^2+^, Ca^2+^, Mg^2+^, Al^3+^, Fe^2+^, and Ga^3+^, immediately after the addition of each ion and mixing the solution. The results are shown in Figure 2. The obtained results indicated that Cu^2+^ was the sole cation that contributed to an efficient and rapid fluorescence quenching of all studied peptides. However, in the cases of EHHQY, EYHHQ, and KYHHE (contrary to EHYHQ), the addition of Ni^2+^ ions led to a very slow but indisputable (approximately 6%) decrease in the fluorescence intensity. We are using this as an evidence for very slow binding of Ni^2+^ ions to EHHQY, EYHHQ, and KYHHE but, at the same time, not to EHYHQ. Indeed, additional spectrophotometric experiments revealed that EHYHQ (in contrast to EHHQY, EYHHQ, and KYHHE) does not interact with Ni^2+^ ions (Figure S2 in the Supplementary Materials). Hence, an exceptional, significant (approximately 3.5 times in the presence of 50 μM of metal ion) and very fast (few seconds) decrease in the fluorescence intensity of EHYHQ caused only by Cu^2+^ ions indicated its specific sensing ability to these cations. It is worth to mention that (similarly to absorbance measurements) an increase in the fluorescence quenching was observed until EHYHQ was saturated at approximately 0.5 molar equivalent of added Cu^2+^, and subsequent addition of the metal ion produced no further quenching (details will be described further and shown in Figure 3). One of the most probable reasons responsible for the observed phenomenon is the formation of a nonfluorescent Cu^2+^-peptide complex due to a chelation enhancement quenching effect [33] or/and ligand to metal charge transfer [34,35,36]. The presence of a paramagnetic d^9^ Cu^2+^ ion in the proximity of a tyrosine residue makes that, in each complex, the forbidden intersystem crossing becomes faster (paramagnetic effect) [17,33]. As a result, the studied complexes after excitation undergo intersystem crossing from S_1_ to T_1_ state of the fluorophore that is further deactivated by bimolecular non-radiative processes.

UV absorption and fluorescence titration experiments of EYHHQ, EHYHQ, EHHQY, and KYHHE (0.1 mM) with Cu^2+^ ions (0–80 µM) in 5 mM MES buffer (pH 6.0) were also carried out in order to establish the binding stoichiometry of the formed complexes as well as to determine appropriate stability constant (*K*) values. The binding stoichiometry of the resulting complexes was evaluated according to commonly used Yoe and Jones’ method [37]. According to this method, the analytical signals measured (in our experiment, these are (i) $\frac{F_{0}\;-\;F}{F_{0}}$, where F_0_ denotes fluorescence intensity (measured at 305 nm, λ_ex_ = 275 nm) of the peptide in the absence of Cu^2+^ ions while F denotes fluorescence intensity (measured at 305 nm, λ_ex_ = 275 nm) of the peptide at any given concentration of Cu^2+^ ions, and (ii) absorbance measured at 275 nm) were plotted versus the corresponding molar concentration ratio $\frac{c_{{\mathrm{Cu}}^{2+}}}{c_{\mathrm{peptide}}}$, and appropriate curves were obtained. Subsequently, the stoichiometry of the complexes was estimated from the points where these curves changed their slopes (Figure 3). As these results revealed unequivocally that the complexes of the stoichiometry of 1:2 (Cu^2+^ to peptide) are formed for all the investigated systems, the equilibrium model presented in Table 1 was applied to calculate the stability constants of the complexes under study using the HypSpec program [38].

**Figure 4:**
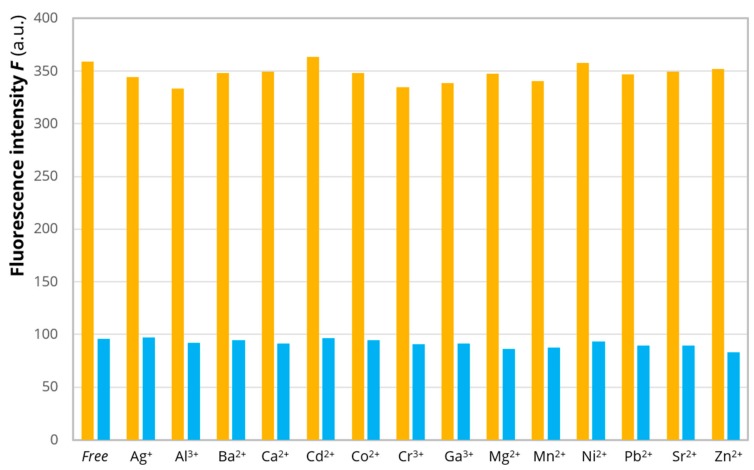
The fluorescence intensity (measured at 305 nm) of EHYHQ (0.1 mM) with the addition of 0.25 mM of various metal ions (orange columns) in 5 mM MES buffer (pH 6.0) and then with the addition of 0.05 mM of Cu^2+^ ions (blue columns): Molar ratio 1:2.5:0.5 (EHYHQ to Me^n+^ to Cu^2+^).

To estimate the sensing ability and selectivity of EHYHQ toward Cu^2+^ ions, we have carried out competitive experiments, where the peptide was exposed to various metal cations separately (Mn^2+^, Co^2+^, Ni^2+^, Zn^2+^, Cr^3+^, Cd^2+^, Ag^+^, Pb^2+^, Sr^2+^, Ba^2+^, Ca^2+^, Mg^2+^, Al^3+^, and Ga^3+^) and then to Cu^2+^ ions (Figure 4). The fluorescence of 0.1 mM of EHYHQ in the 5 mM MES buffer was firstly evaluated in the absence of any ions, then in the presence of each potentially interfering ion (0.25 mM), and finally in the presence of a mixture of potentially interfering ion (0.25 mM) and Cu^2+^ (0.05 mM). The obtained results revealed that only Cu^2+^ ions induced a significant decrease in the fluorescence intensity of EHYHQ at 305 nm, while under the action of other ions (used at concentrations five times higher than Cu^2+^ ions), there were no changes observed or the fluorescence quenching was negligible. Furthermore, the decrease in the fluorescence intensity of EHYHQ in the presence of a mixture of Cu^2+^, Mn^2+^, Fe^2+^, Co^2+^, Ni^2+^, and Zn^2+^ (each 80 μM) was comparable (within the range of experimental error) with that observed for 80 μM of Cu^2+^ ions alone (Figure S3 in the Supplementary Materials). All these results demonstrated the good anti-interference ability of EHYHQ to other cations. It consequently permitted highly selective detection and determination of Cu^2+^ ions in aqueous solutions with the use of that peptide.

**Figure 5:**
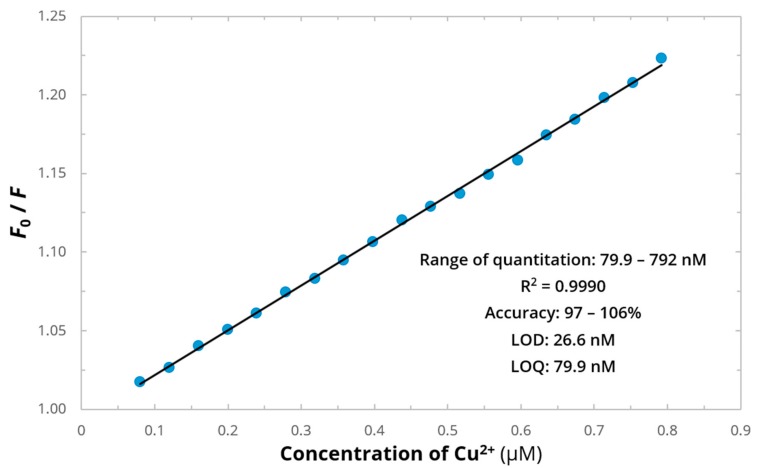
A calibration plot reflecting the relationship between the relative fluorescence decrease of EHYHQ (0.1 mM) and the concentration of Cu^2+^ ions: the inset gathers analytical characteristics of the graph. $\frac{F_{0}}{F}$ values are shown as a mean value from 30 data points (*n* = 30). LOD—limit of detection; LOQ—limit of quantification.

Figure 5 shows the linear plot of $\frac{F_{0}}{F}$ for EHYHQ as a function of the concentration of Cu^2+^ ions that fits the Stern–Volmer equation, suggesting that only one type of quenching (either dynamic or either static) is involved in the fluorescence intensity decrease. It revealed that the extent of the fluorescence quenching is proportional to the concentration of Cu^2+^ ions over the range of 79.9–792 nM, which forms the basis of the method for the Cu^2+^ determination. The inset of Figure 5 tabulates the analytical parameters obtained for the calibration graph reflecting the determination of Cu^2+^ ions with the use of EHYHQ as a fluorescent sensor. In the presence of EHYHQ as a ligand, the limit of detection (LOD) of Cu^2+^ was estimated to be less than 27 nM, which is ∼750 times lower than the recommended maximum contaminant level for these cations in drinking water [6]. Within the investigated range of quantitation, a linear relationship was observed (between the relative fluorescence decrease and the concentration of Cu^2+^ ions)—as it is evidenced by the values of the coefficients of determination (R^2^) close to unity. Furthermore, the determination of Cu^2+^ ions with the use of EHYHQ is characterized with a high accuracy.

The determination of fluorescence lifetimes was carried out to confirm that the static quenching is solely responsible for the observed fluorescence decrease. According to performed time-resolved experiments in the case of all peptides under study, the values of their fluorescence lifetimes were constant regardless of the Cu^2+^ ions concentration and equal to 1.12 ns for EHHQY, 0.78 ns for EHYHQ, 0.85 ns for EYHHQ, and 0.77 ns for KYHHE. Therefore, since Cu^2+^ ions had no effect on fluorescence lifetimes of all studied peptides, it could be concluded that the mechanism of fluorescence quenching is purely static. It is in a great agreement with the results shown in Figure 1, as appropriate complex formation very often impacts the absorption spectrum of the fluorophore.

**Figure 6:**
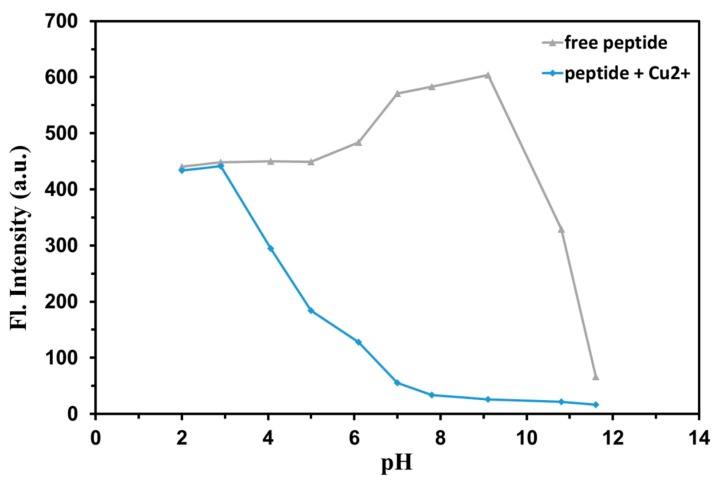
The pH influence on the fluorescence intensity of EHYHQ (0.1 mM) in the absence and presence of 80 µM of Cu^2+^.

The pH dependence of the fluorescence emission intensities of EHYHQ (0.1 mM) in the absence and presence of 80 µM of Cu^2+^ ions was examined over a wide range of pH values. As shown in Figure 6, the determination of Cu^2+^ ions can work well in the range of pH approximately 4.0–11.0, where the decrease of fluorescence intensity of EHYHQ under the action of Cu^2+^ ions is significant. These observations indicate that EHYHQ is a very effective fluorescent sensor for Cu^2+^ ions under the environmental and physiological conditions.

The reversibility of EHYHQ towards Cu^2+^ ions was evaluated as shown in Figure S4 in the Supplementary Materials. When EHYHQ (0.1 mM, pH 7.4) is saturated by the addition of Cu^2+^ ions (0.5 equiv.), approximately 3 times decrease in fluorescence intensity is observed. However, upon the addition of Cu^2+^ chelating agent (2 equiv. of EDTA) to the EHYHQ with Cu^2+^ solution, a significant increase in the fluorescence intensity was observed. The obtained results clearly demonstrate that Cu^2+^ ions can be effectively replaced by EDTA from Cu^2+^-EHYHQ complex and consequently prove that Cu^2+^ ions recognition is a complexing and reversible process. 

**Figure 7:**
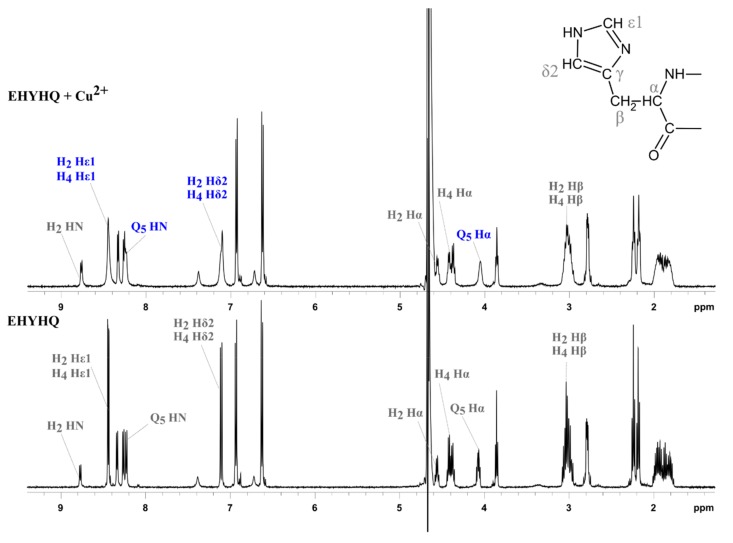
^1^H NMR spectrum of 8 mM EHYHQ in the presence of Cu^2+^ ions in 2:1 molar ratio (upper panel) and in the absence of Cu^2+^ ions (lower panel) along with selected signals identities: The most significantly broadened signals are marked with blue.

In order to propose the mechanism of interactions between EHYHQ and Cu^2+^ ions, we have performed additional NMR spectroscopy investigations. Figure 7 shows the effect of the presence of Cu^2+^ ions on the appearance of the ^1^H NMR spectrum of EHYHQ. We have observed that the addition of Cu^2+^ ions to the peptide solution caused no significant change of linewidths and chemical shifts except for aromatic protons of the imidazole ring, but slight line-broadening was observed. This is in good agreement with reports [39,40] that paramagnetic Cu^2+^ ions are known to broaden ^1^H NMR signals without any dramatic change in chemical shifts when the ^1^H is in close vicinity to a cation or on a donor atom directly coordinated to a metal. As the nitrogen atom of the imidazole ring very effectively competes with the terminal amino group as an anchoring point for Cu^2+^, it strongly points to histidine residues as anchoring sites and to their involvement in complexation [41,42]. As the signals corresponding to protons of glutamine are slightly broadened too, it may demonstrate a participation of that residue (along with histidine) in a complexation as well [43]. Taking into consideration the fact that Ni^2+^ ions unambiguously interact with EHHQY, EYHHQ, and KYHHE but, at the same time, not with EHYHQ (Figure S2 in the Supplementary Materials), we could assume that both histidine residues placed side by side in a peptide sequence might be responsible for binding properties in the case of that cation. On the other hand, the tyrosine residue placed between histidine residues in the EHYHQ sequence has no influence on the interactions between that peptide and Cu^2+^ ions. A visualization of predicted structure of complex between EHYHQ and Cu^2+^ (both *cis* and *trans* isomers) is presented in Figure S5 in the Supplementary Materials.

**Figure 8:**
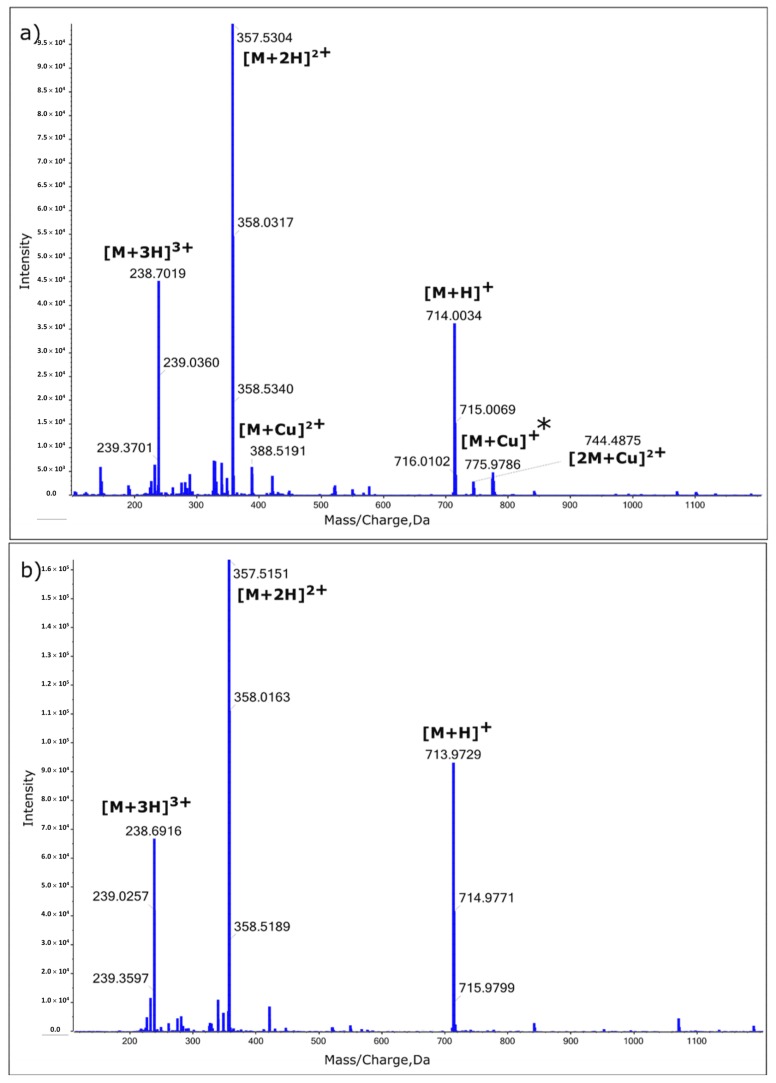
The MS spectra of the 2 mM solutions of EHYHQ in the presence of 10 mM of Cu^2+^ ions (**a**) and in the absence of Cu^2+^ ions (**b**). * Cu(II) can be reduced to Cu(I) in positive-ion mode electrospray mass spectrometry [44,45].

In order to confirm the type of interactions and stoichiometry of the complex formed between EHYHQ and Cu^2+^ ions, additional MS investigations were performed—EHYHQ in the absence of Cu^2+^ and in the presence of Cu^2+^ in ratio 1:5 (peptide to Cu^2+^) were analyzed using a 2 mM solution in water (Figure 8, Figures S6–S8 in the Supplementary Materials, and Table S1 in the Supplementary Materials). The spectrum of pure EHYHQ revealed singly and doubly charged molecular ions with *m/z* 713.9728 ([M + H]^+^) and 357.5150 ([M + 2H]^2+^), respectively. Further, the analysis of the EHYHQ–Cu^2+^ solution (molar ratio 1:5) showed additionally (apart from the signals of [M + H]^+^ and [M + 2H]^2+^) molecular ions with *m/z* 775.9771 ([M + Cu]^+^), 744.4875 ([2M + Cu]^2+^), and 388.5192 ([M + Cu]^2+^), respectively. This is in line with the results obtained from the UV and fluorescence spectroscopy measurements and confirms the fact that EHYHQ under experimental conditions binds Cu^2+^ ions in 2:1 molar.

The analytical performance of the proposed Cu^2+^ ion sensor was compared with some other reported selective sensors in related literature, and the results are presented in Table 2. Our sensor showed very good LOD (comparable or lower with some other recently reported fluorescent sensors), no interference by other competitive metal ions, instant response time, and wide pH range. The analytical results indicate that proposed peptide is a simple and highly sensitive fluorescent sensor for selective determination of Cu^2+^ ions in aqueous samples.

## 3. Materials and Methods 

### 3.1. Materials

The chosen pentapeptides (EYHHQ, EHYHQ, EHHQY, and KYHHE) were synthesized using a procedure described previously [51] and were then chromatographically purified using gradient elution involving a water and a 10% to 100% (v/v) acetonitrile containing 0.1% (v/v) trifluoroacetic acid mixture in a 3.5 μm, 3.0 mm × 100 mm XBridgeShield RP18 column (purchased from Waters, Milford, MA, USA). The products were then characterized by mass spectrometry and an absorption peak at 214 nm. Appropriate chromatograms and mass spectra confirming the purity and the identity of the investigated peptides are presented in Figures S9–Figure S12 in the Supplementary Materials. The concentration of the studied peptides was confirmed spectrophotometrically based on the absorbance and the value of molar extinction coefficient of tyrosine determined at 280 nm (ε = 1280 M^−1^cm^−1^) [52]. 2-(*N*-Morpholino)ethanesulfonic acid (MES) and the nitrate and chloride salts of the chosen metal cations (Cu^2+^, Mn^2+^, Co^2+^, Ni^2+^, Zn^2+^, Cr^3+^, Cd^2+^, Ag^+^, Pb^2+^, Sr^2+^, Ba^2+^, Ca^2+^, Mg^2+^, Al^3+^, Fe^2+^, and Ga^3+^) were purchased from Sigma Aldrich (Poland; analytical grade or the highest purity available) and used without further purification. The solutions were prepared using ultra purified water (a conductivity not exceeding 0.18 μS/cm) deionized by a Milli-QSP reagent water system (Merck Millipore, Burlington, MA, USA). 

### 3.2. Methods

#### 3.2.1. UV Absorption Spectroscopy

UV absorption spectra of EYHHQ, EHYHQ, EHHQY, and KYHHE (0.1 mM) with several 80 µM metal cations (Cu^2+^, Mn^2+^, Co^2+^, Ni^2+^, Zn^2+^, Cr^3+^, Cd^2+^, Ag^+^, Pb^2+^, Sr^2+^, Ba^2+^, Ca^2+^, Mg^2+^, Al^3+^, Fe^2+^, and Ga^3+^) were acquired at room temperature using a Perkin Elmer Lambda 650 UV/Vis spectrophotometer (Waltham, MA, USA) with a 1.0 cm quartz cell. The measurements were conducted in 5 mM MES buffer (pH 6.0). In performing UV absorption titration experiments, 2 mL of each 0.1 mM peptide solution was titrated with twenty 1 μL aliquots of Cu^2+^ solution at 8 mM to achieve 1:0.8 peptide to metal ion molar ratio. Absorbance was measured at 275 nm.

#### 3.2.2. Steady-State and Time-Resolved Fluorescence Spectroscopy

Fluorescence emission spectra (λ_ex_ = 275 nm) of EYHHQ, EHYHQ, EHHQY, and KYHHE (0.1 mM) with several 80 µM metal cations (Cu^2+^, Mn^2+^, Co^2+^, Ni^2+^, Zn^2+^, Cr^3+^, Cd^2+^, Ag^+^, Pb^2+^, Sr^2+^, Ba^2+^, Ca^2+^, Mg^2+^, Al^3+^, Fe^2+^, and Ga^3+^) as well as all steady-state fluorescence intensity measurements were performed at room temperature on a Cary Eclipse Varian spectrofluorometer (Agilent, Santa Clara, CA, USA) equipped with a 1.0 cm quartz cell. In performing fluorescence titration experiments, 2 mL of each peptide solution at 0.1 mM was titrated with twenty 1 μL aliquots of Cu^2+^ solution at 8 mM to achieve 1:0.8 peptide to metal ion molar ratio. Decreases in the fluorescence intensity were measured at 305 nm, corresponding to the maximum of the peptide emission [53]. Possible absorption of light by the added cations at the excitation and emission wavelengths of peptides was measured. The influence of an inner filter effect was estimated on the basis of the following Equation (1):

(1) $$F_{\mathrm{corr}}=F_{\mathrm{obs}}\times 10^{\frac{A_{\mathrm{ex}}+A_{\mathrm{em}}}{2}}$$

where F_corr_ and F_obs_ denote the corrected and observed fluorescence intensities, respectively, while A_ex_ + A_em_ is the sum of the absorbance of the peptide and ion measured at the excitation and emission wavelengths, respectively [54].

Time-resolved fluorescence experiments were conducted with a FluoTime 300 high-performance fluorescence lifetime spectrometer (PicoQuant, Berlin, Germany) at 20 °C. The probes were excited with the use of a pulsed LED of the PLS series (λ_ex_ = 287 nm). The fluorescence lifetimes of all peptides (0.1 mM) were determined in the absence and presence of increasing concentrations of Cu^2+^ ions up to 80 µM at the wavelength corresponding to the maximum of the peptide emission (λ_em_ = 305 nm).

Both steady-state and time-resolved experiments were conducted in 5 mM MES buffer (pH 6.0).

#### 3.2.3. Analytical Procedure

The limit of quantification (LOQ) was evaluated as the lowest point of a linear calibration plot (the plot of F_0_/F as a function of the concentration of Cu^2+^ ions) obtained with precision of <10% RSD and accuracy between 90 and 110%, while limit of detection (LOD) was estimated as LOQ divided by three [55].

#### 3.2.4. NMR Spectroscopy

The ^1^H-NMR spectra of 8 mM EHYHQ alone and a mixture of 8 mM EHYHQ and 4 mM Cu^2+^ (a 2:1 peptide to Cu^2+^ ions stoichiometric molar ratio was obtained from absorbance and fluorescence intensity measurements) were recorded at 25 °C on a Varian Unity Inova 500 (500 MHz) spectrometer (Agilent, Crawley, UK) at the Intercollegiate NMR Laboratory at the Technical University of Gdańsk (Poland) and analyzed with the SPARKY program [56]. The samples were dissolved in 90% H_2_O/10% D_2_O.

#### 3.2.5. Liquid Chromatography Coupled with Mass Spectrometry (LC-MS)

Liquid chromatographic analyses were performed on an ultrahigh-performance liquid chromatography system Nexera (Shimadzu, Kyoto, Japan). Chromatographic conditions were as follows: a Kinetex column (Phenomenex, 1.7 µm, C18, 100 Å, 2.1 × 150 mm), a flow rate of 0.3 mL · min^−1^, and a gradient elution with 80% acetonitrile and water (from 0% to 40% acetonitrile). The column temperature was maintained at 25 °C. The effluent was diverted to waste for 2 min after injection during each analysis. The liquid chromatographic system was coupled directly to a TripleTOF 5600+ (SCIEX, Framingham, MA, USA) mass spectrometer. Mass spectrometry was operated in positive mode at 200 °C with a needle voltage of 3000 V and with nitrogen as curtain gas and nebulizer gas. The declustering potential was 30 V, and the collision energy was 7 V.

## 4. Conclusions

We have investigated the influence of the position of a tyrosine residue in selected peptides sequence on the binding properties with various transient metal ions. Among the four peptides studied, EHYHQ can selectively and sensitively detect Cu^2+^ ions over other common metal cations (Mn^2+^, Co^2+^, Ni^2+^, Zn^2+^, Cr^3+^, Cd^2+^, Ag^+^, Pb^2+^, Sr^2+^, Ba^2+^, Ca^2+^, Mg^2+^, Al^3+^, Fe^2+^, and Ga^3+^) in the MES buffer, leading to a significant fluorescence quenching. The limit of detection of Cu^2+^ ions with the use EHYHQ was found to be approximately 27 nM. The binding stoichiometry of the resulting complexes with Cu^2+^ was evaluated, and their stability constant values were determined. Finally, the possible mechanism of the interactions between EHYHQ and Cu^2+^ ions was proposed.

## Figures and Tables

**Table 1:** log*K* values (standard deviation in parentheses) for the Cu^2+^-peptide complexes in 5 mM MES buffer solution (pH 6.0) calculated for T = 25 °C: Cu and P denote the Cu^2+^ ion and the peptide, respectively (the charges are omitted for the sake of clarity). Twenty-two data points were used to estimate each standard deviation (*n* = 22).

Reaction	log*K_n_*	Cu^2+^-EYHHQ	Cu^2+^-EHYHQ	Cu^2+^-EHHQY	Cu^2+^-KYHHE
Cu + P = CuP	log*K*_1_	1.48 (±0.03)	1.25 (±0.01)	1.55 (±0.01)	1.46 (±0.01)
CuP + P = CuP_2_	log*K*_2_	3.34 (±0.08)	3.25 (±0.02)	3.34 (±0.02)	2.55 (±0.05)
Cu + 2P = CuP_2_	log*K*	4.82 (±0.04)	4.50 (±0.01)	4.89 (±0.01)	(±0.01)

**Table 2:** Comparison of some recent fluorescent sensors for Cu^2+^ ions.

Reference	LOD	Operation System	Reversibility	pH
[17]	120 nM	ON-OFF (HEPES buffer)	-	5.0–8.0
[46]	23.5 nM	ON-OFF (HEPES buffer)	yes	6.0–12.0
[34]	10.9 nM	ON-OFF (PBS buffer)	-	5.0–8.0
[47]	40.4 nM	OFF-ON (H_2_O:CH_3_CN, 1:9 v/v)	-	5.0–7.0
[48]	15 nM	ON-OFF (HEPES buffer)	yes	7.0–12.0
[49]	1 μM	ON-OFF (EtOH)	-	-
[50]	0.55–0.62 μM	ON-OFF (phosphate buffer)	-	7.0–11.0
This work	26.6 nM	ON-OFF (MES buffer)	yes	4.0–11.0

The symbol “-“ denotes that parameter was not tested or mentioned.

## Supplementary Materials

Supplementary materials can be found at https://www.mdpi.com/1422-0067/21/3/743/s1.
