# Supplementary material for: A Pentapeptide with Tyrosine Moiety as Fluorescent Chemosensor for Selective Nanomolar-Level Detection of Copper(II) Ions
Source: Int J Mol Sci. 2020 Jan 23;21(3):743. doi: 10.3390/ijms21030743 (PMC7037753; doi:10.3390/ijms21030743)
Supplement: Supplementary file 1 [file ijms-21-00743-s001.pdf]

International Journal of *Molecular Sciences*

Article – Supplementary Materials

A Pentapeptide with Tyrosine Moiety as Fluorescent Chemosensor for Selective Nanomolar-Level Detection of Copper(II) Ions

Krzysztof Żamojć [1,*], Dominik Kamrowski [1], Magdalena Zdrowowicz [1], Dariusz Wyrzykowski [1], Wiesław Wiczk [1], Lech Chmurzyński [1] and Joanna Makowska [1]

[1] Faculty of Chemistry, University of Gdańsk, Wita Stwosza 63, 80-308 Gdańsk, Poland
* Correspondence: krzysztof.zamojc@ug.edu.pl; Tel.: +48-58-523-50-57

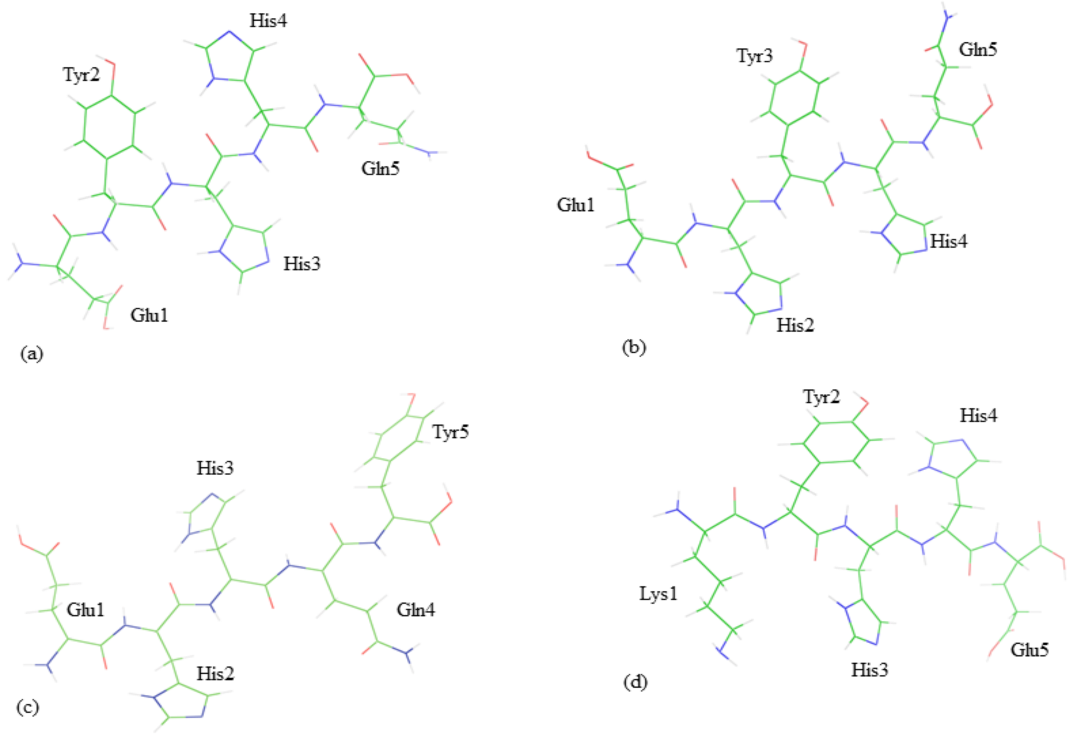

Figure S1. A visualization of EYHHQ (a), EHYHQ (b), EHHQY (c), and KYHHE (d).

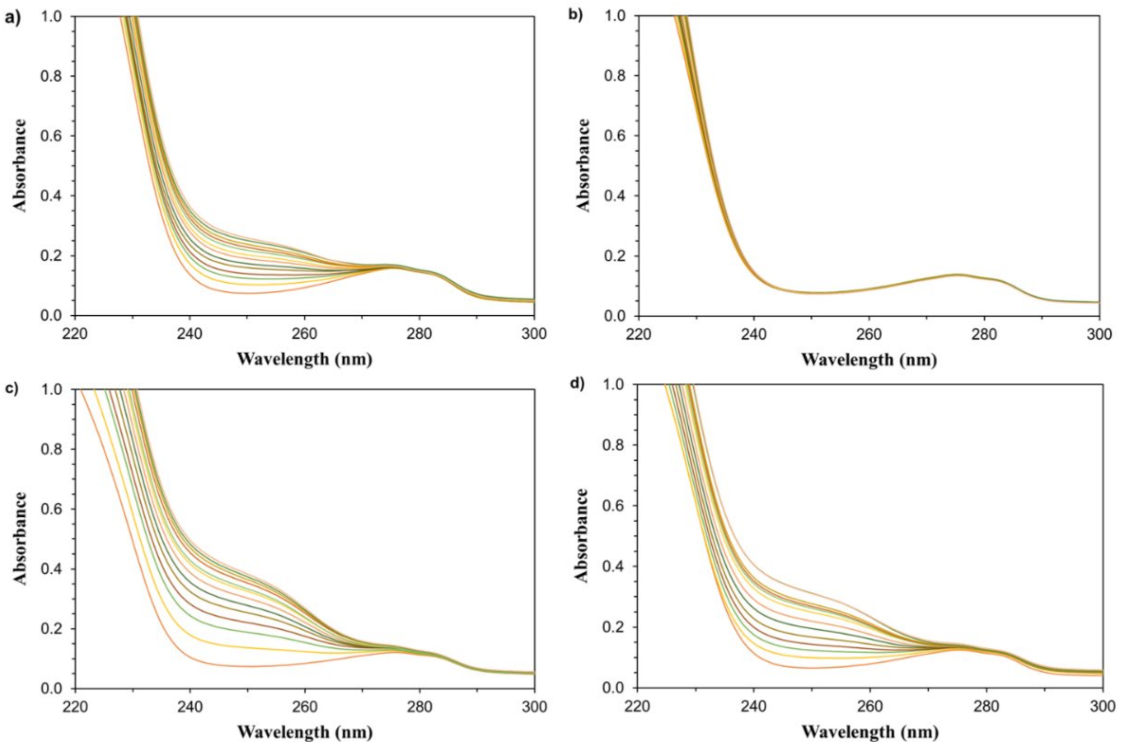

Figure S2. UV absorption spectra of EYHHQ (a), EHYHQ (b), EHHQY (c), and KYHHE (d) (0.1 mM in 5 mM MES buffer, pH 6.0) in the presence of different concentrations (0 – 95 μM) of Ni^{2+}. Spectrum with the lowest absorbance on each graph corresponds to the peptide in the absence of Ni^{2+} ions, while the spectrum with the highest absorbance on each graph corresponds to the peptide in the presence of 95 μM of Ni^{2+}. All the spectra were registered 7 days after the addition of Ni^{2+} ions.

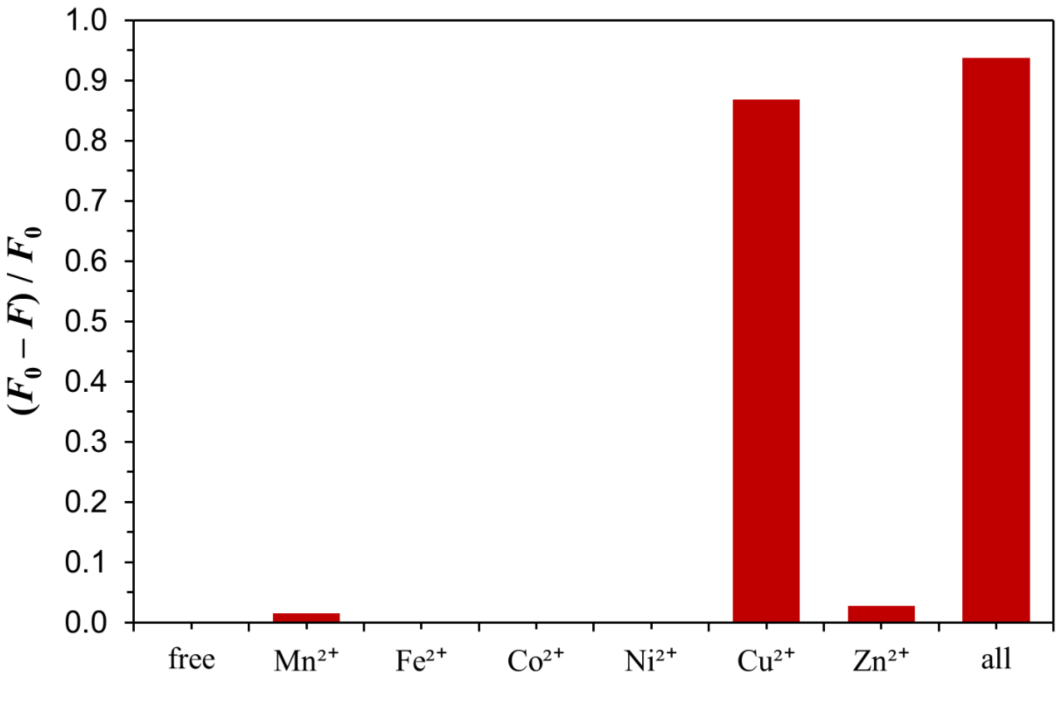

Figure S3. $\frac{F_0-F}{F_0}$ of EHYHQ (0.1 mM in 5 mM MES buffer, pH 6.0) in the presence of 80 μM of various metal ions (Cu^{2+}, Mn^{2+}, Fe^{2+}, Co^{2+}, Ni^{2+}, and Zn^{2+}) and in the presence of a mixture of all these ions (each 80 μM). F_0 – fluorescence intensity (measured at 305 nm, λ_{ex} = 275 nm) of the peptide in the absence of

any ions; F – fluorescence intensity (measured at 305 nm, λ_{ex} = 275 nm) of the peptide in the presence of 80 µM of each ion or mixture of ions.

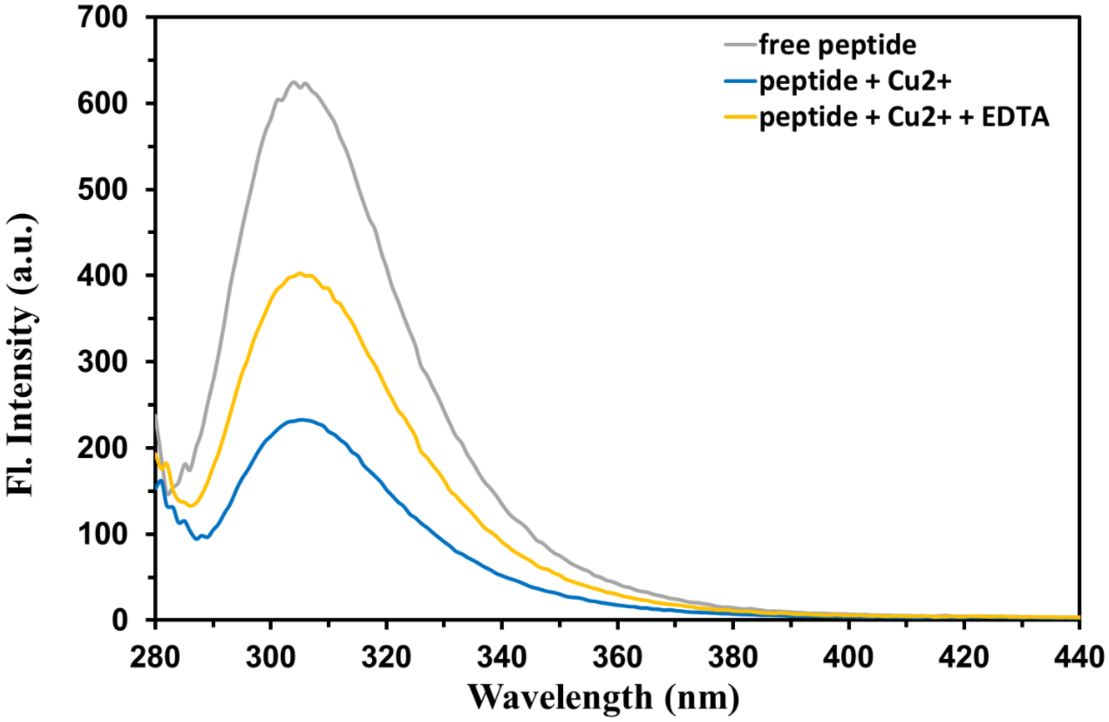

Figure S4. The fluorescence emission spectra of EHYHQ (0.1 mM, pH 7.4) upon subsequent addition of Cu^{2+} (0.5 equiv.) and then EDTA (2 equiv.).

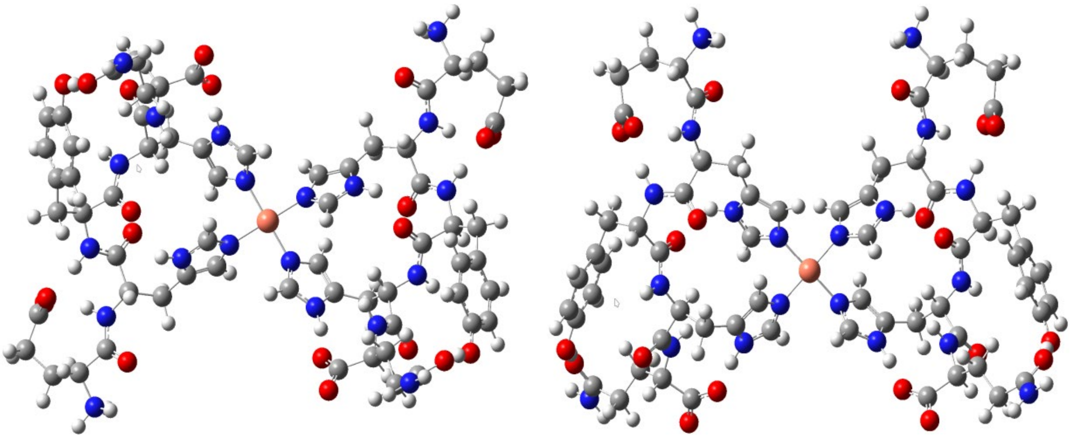

Figure S5. A visualization of predicted structure of complex between EHYHQ and Cu^{2+} (isomer *trans* on the left and isomer *cis* on the right).

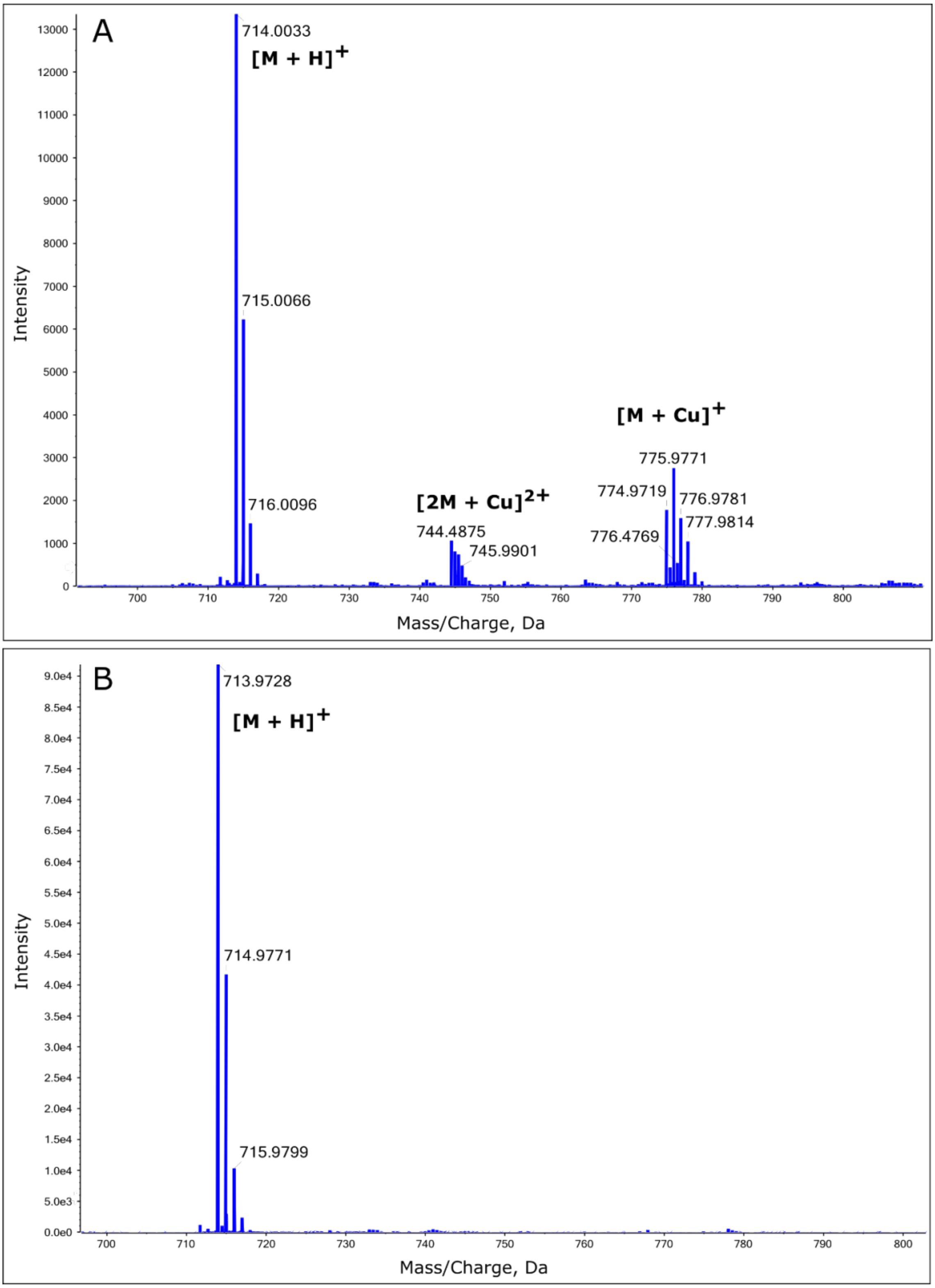

Figure S6. Magnification (*m*/*z* range approximately 700-800) of MS spectra presented in Figure 8.

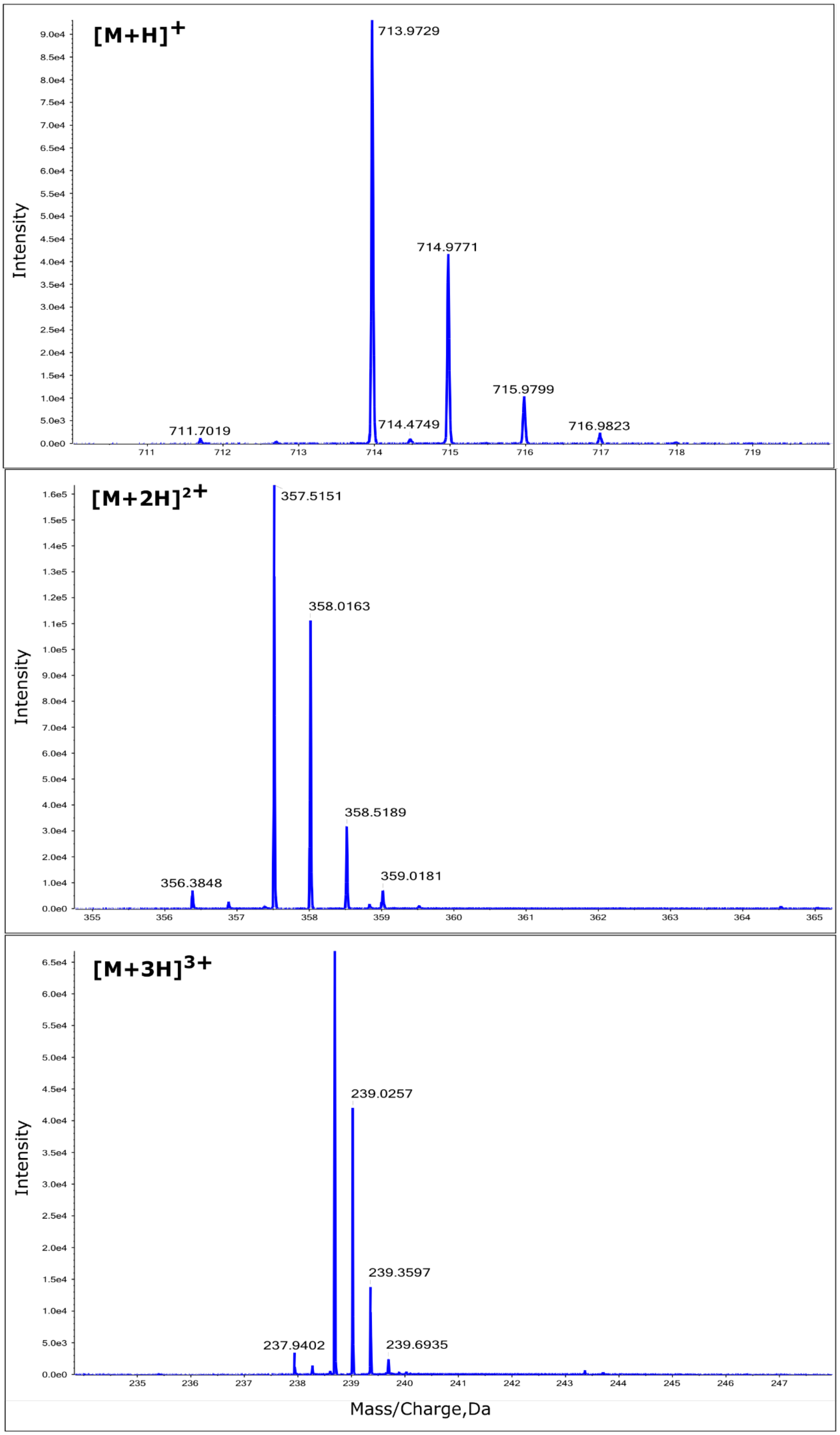

Figure S7. Isotope patterns of identified signals: $[M+H]^{+}$, $[M+2H]^{2+}$, $[M+3H]^{3+}$.

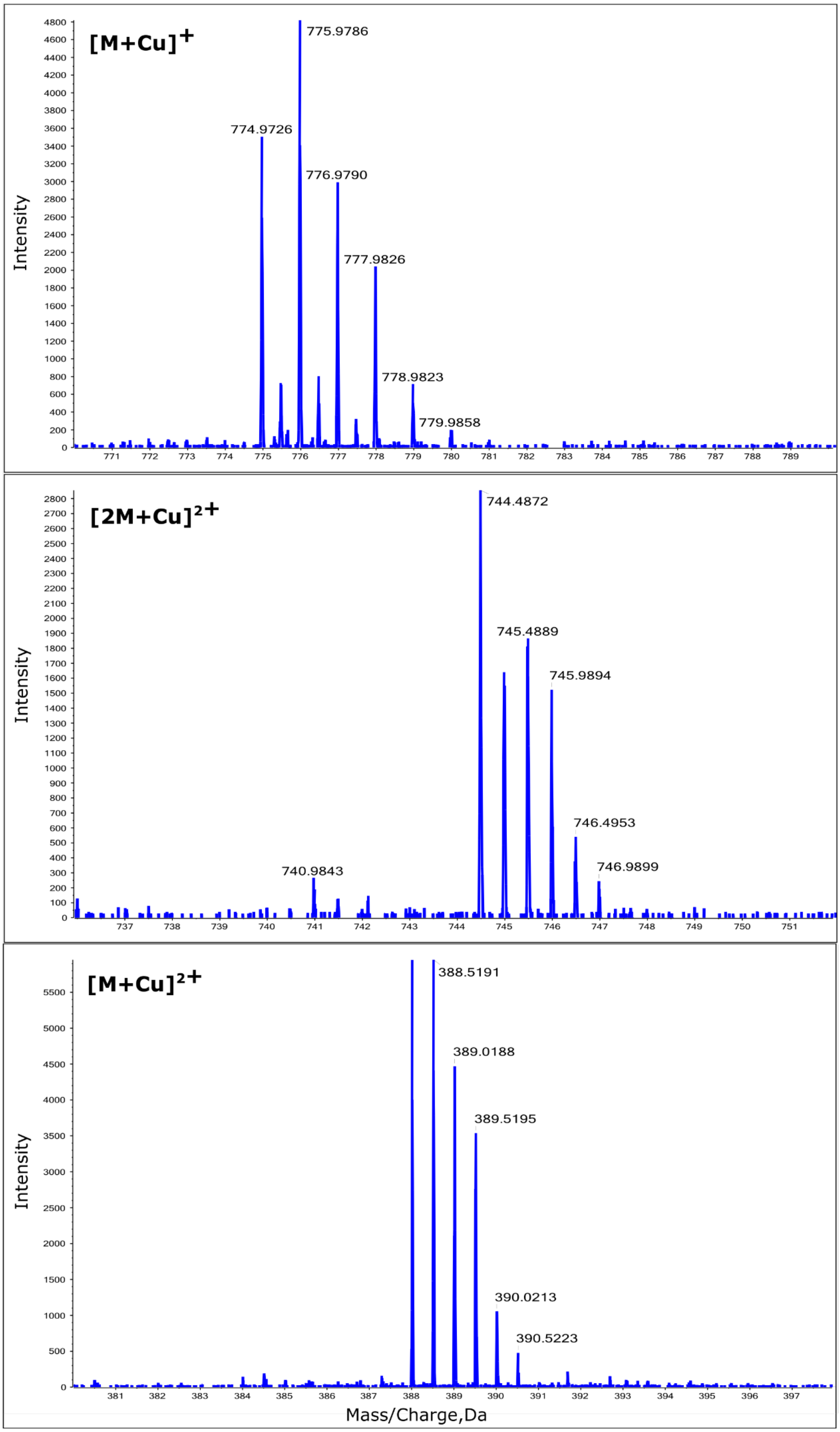

Figure S8. Isotope patterns of identified signals: $[M+Cu]^{+}$, $[2M+Cu]^{2+}$, $[M+Cu]^{2+}$.

Table S1. Measured and calculated masses of ion identities (see Figure 8).

**Ion identity**	**Calculated mass [Da]**	**Measured mass [Da]**
$[M+H]^{+}$	713.3002	713.9729
$[M+2H]^{2+}$	357.1501	357.5151
$[M+3H]^{3+}$	238.4378	238.6916
$[M+Cu]^{+}$	776.2287	775.9786
$[M+Cu]^{2+}$	388.1143	388.5191
$[2M+Cu]^{2+}$	744.2608	744.4875

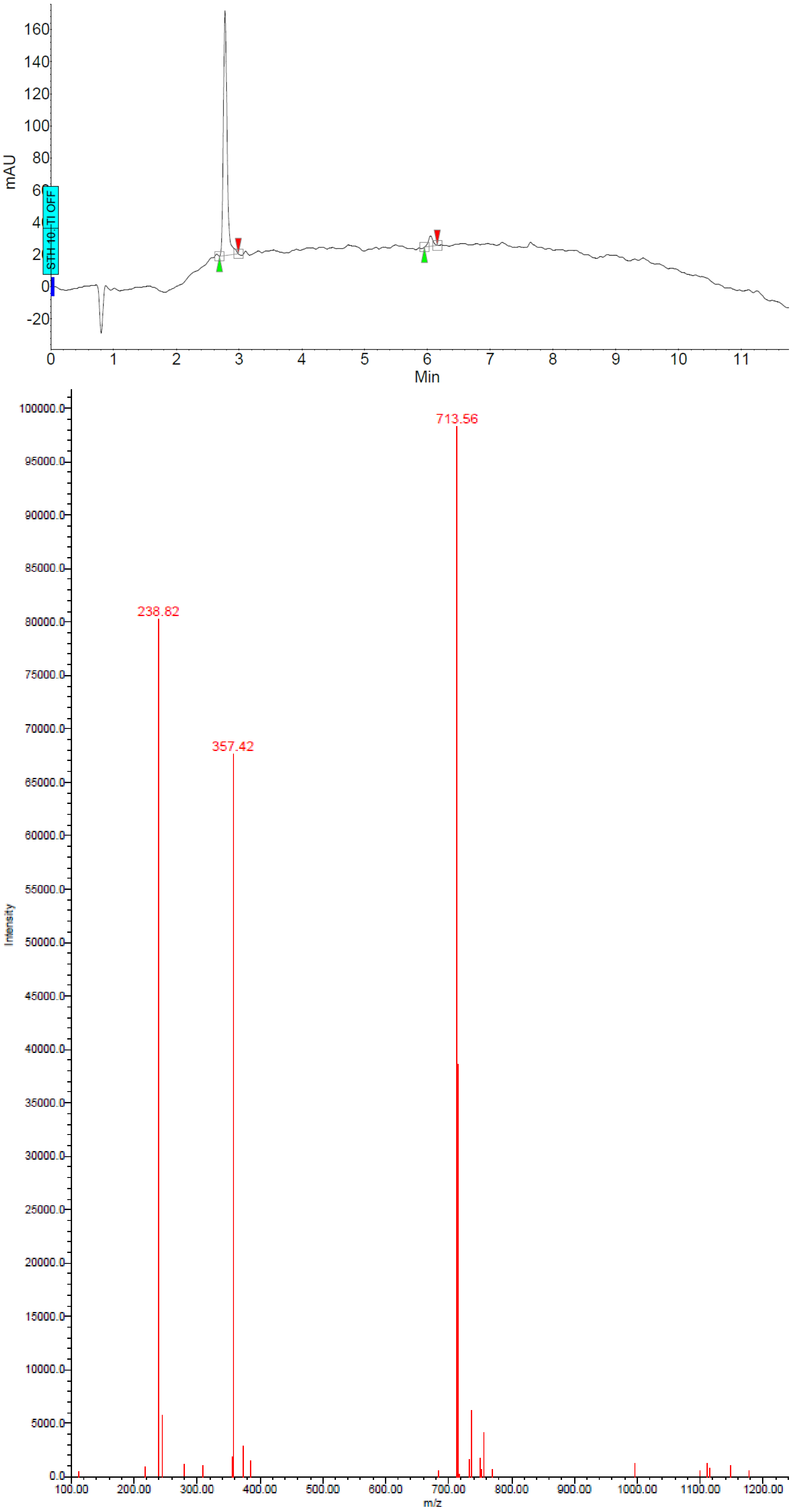

Figure S9. The chromatogram and MS spectrum corresponding to the purity and identity of EYHHQ.

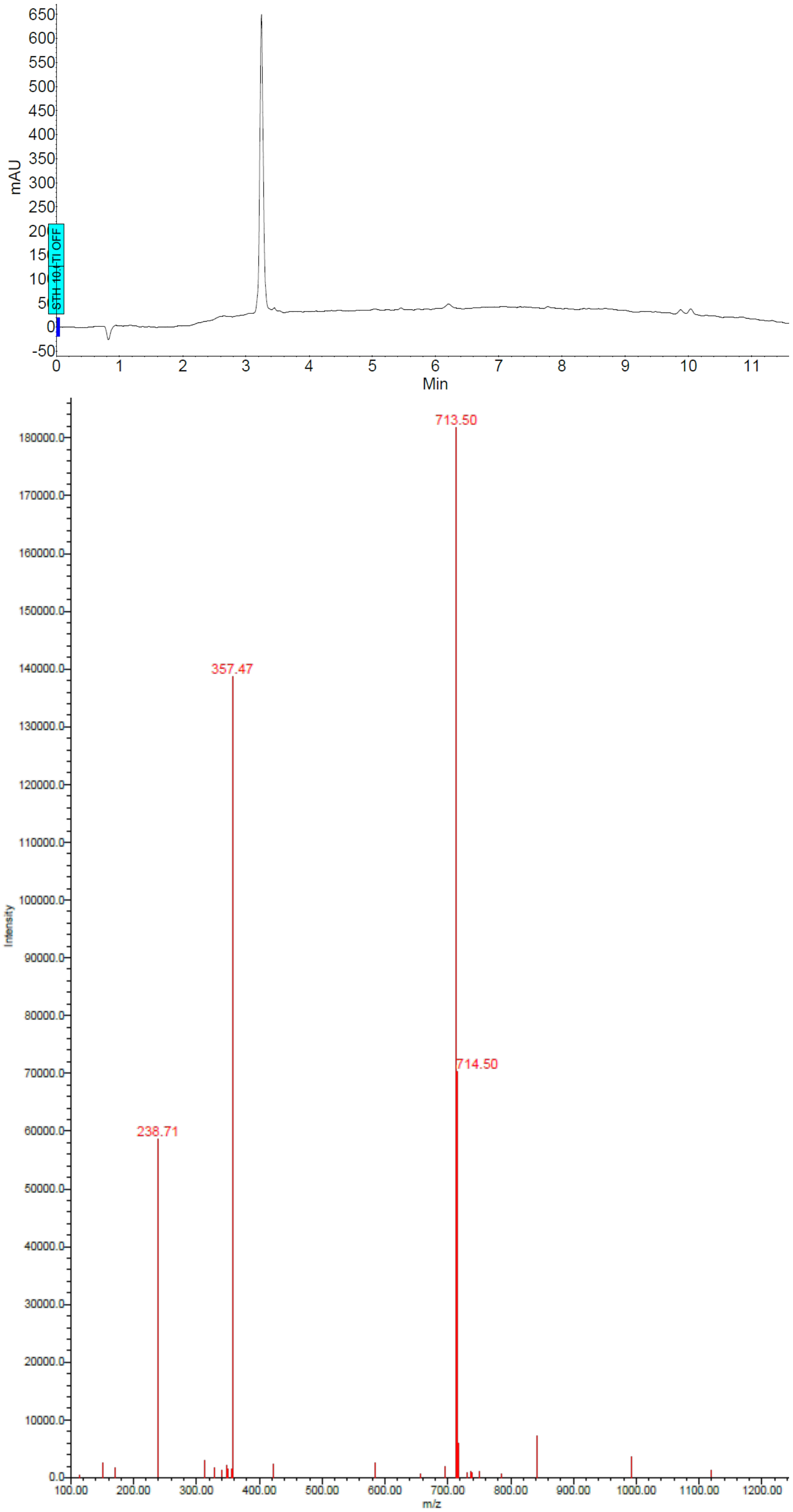

Figure S10. The chromatogram and MS spectrum corresponding to the purity and identity of EHYHQ.

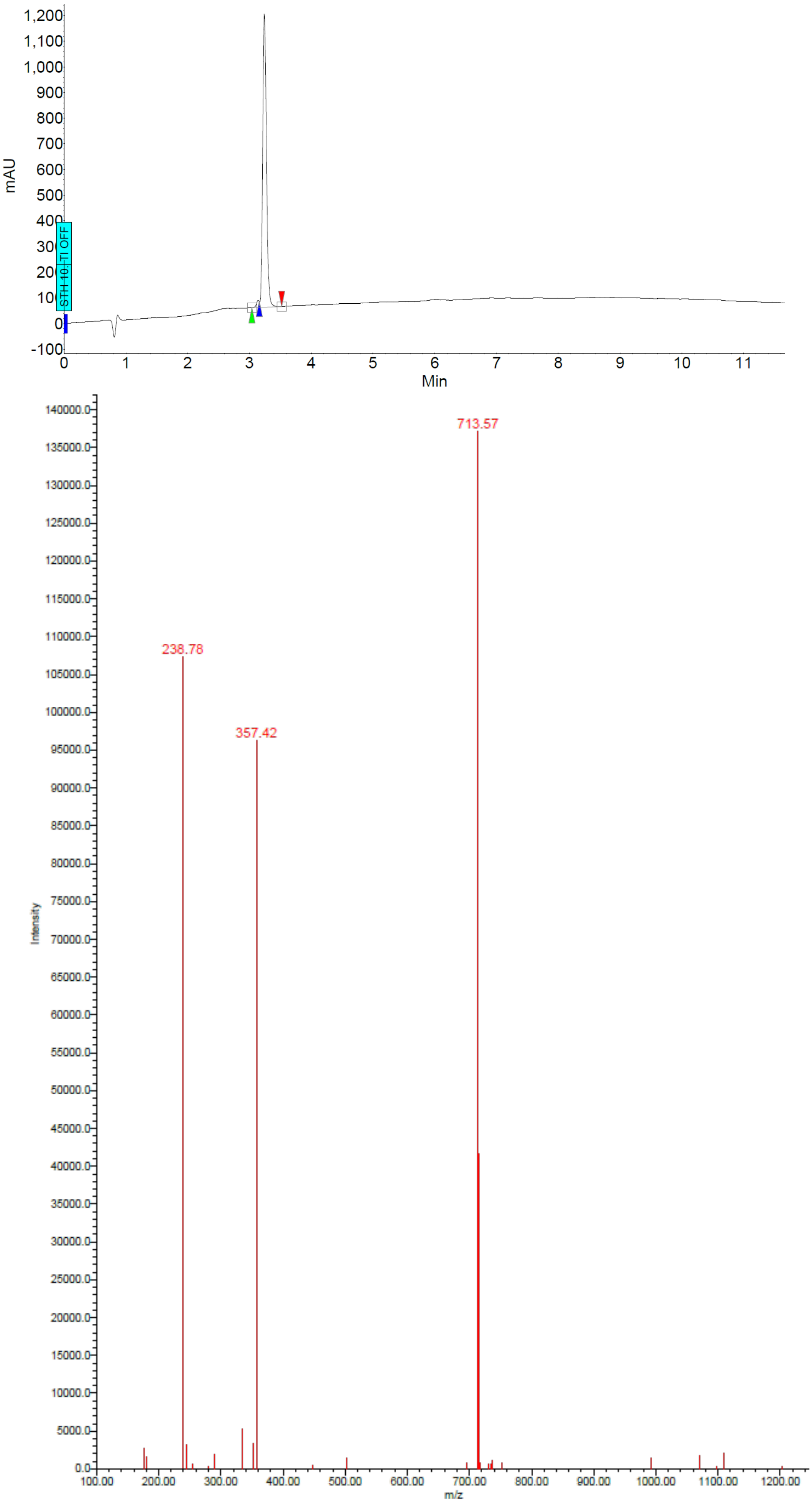

Figure S11. The chromatogram and MS spectrum corresponding to the purity and identity of EHHQY.

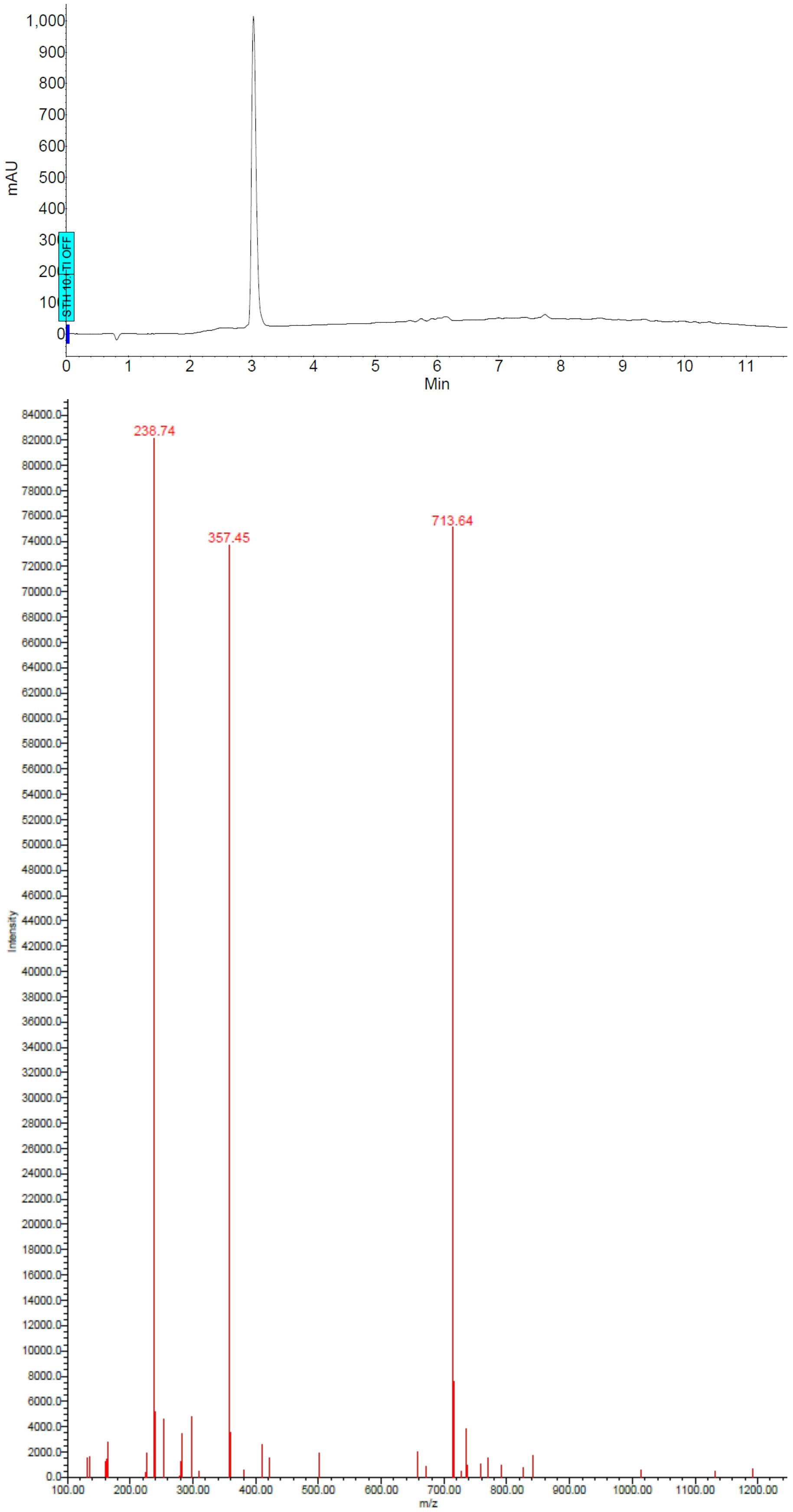

Figure S12. The chromatogram and MS spectrum corresponding to the purity and identity of KYHHE.
